# Detection of condition-specific marker genes from RNA-seq data with MGFR

**DOI:** 10.7717/peerj.6970

**Published:** 2019-05-27

**Authors:** Khadija El Amrani, Gregorio Alanis-Lobato, Nancy Mah, Andreas Kurtz, Miguel A. Andrade-Navarro

**Affiliations:** 1 Berlin Brandenburg Center for Regenerative Therapies (BCRT), Charité—Universitätsmedizin Berlin, Berlin, Germany; 2 Faculty of Biology, Johannes-Gutenberg Universität Mainz, Mainz, Germany

**Keywords:** Marker genes, Gene expression, Tissue specificity, Cell-type specificity, Single cell, RNA-Seq, Transcriptomics, R-package

## Abstract

The identification of condition-specific genes is key to advancing our understanding of cell fate decisions and disease development. Differential gene expression analysis (DGEA) has been the standard tool for this task. However, the amount of samples that modern transcriptomic technologies allow us to study, makes DGEA a daunting task. On the other hand, experiments with low numbers of replicates lack the statistical power to detect differentially expressed genes. We have previously developed MGFM, a tool for marker gene detection from microarrays, that is particularly useful in the latter case. Here, we have adapted the algorithm behind MGFM to detect markers in RNA-seq data. MGFR groups samples with similar gene expression levels and flags potential markers of a sample type if their highest expression values represent all replicates of this type. We have benchmarked MGFR against other methods and found that its proposed markers accurately characterize the functional identity of different tissues and cell types in standard and single cell RNA-seq datasets. Then, we performed a more detailed analysis for three of these datasets, which profile the transcriptomes of different human tissues, immune and human blastocyst cell types, respectively. MGFR’s predicted markers were compared to gold-standard lists for these datasets and outperformed the other marker detectors. Finally, we suggest novel candidate marker genes for the examined tissues and cell types. MGFR is implemented as a freely available Bioconductor package (https://doi.org/doi:10.18129/B9.bioc.MGFR), which facilitates its use and integration with bioinformatics pipelines.

## Introduction

Detection of biomarkers from gene expression datasets, that is, genes particularly expressed in certain samples and not in others, is very useful for distinguishing between different cell types and tissues, as well as for the identificaton of genes with functions specific to those cells and tissues (Forrest et al., 2014). Traditionally, differential gene expression analysis (DGEA) has been the approach of choice for this task, in which pairs of samples are compared with fold changes and *t*-tests. However, next-generation sequencing transcriptomics allow for the study of so many conditions, that the quadratic growth in the number of pairwise comparisons between them quickly makes their analysis tedious and impractical (Cavalli et al., 2011). On the other hand, small sample size experiments lack the adequate power for detecting differential expression (Yu, Fernandez & Brock, 2017). This has led to the development of several methods to pinpoint genes with condition-specific functions. These techniques range from fixed thresholds on the RPKM or FPKM expression values of each sample (Hebenstreit et al., 2011; Wagner, Kin & Lynch, 2013; Will & Helms, 2016), to determination of genes that are significantly expressed in each condition (Kitsak et al., 2016), to information theory- and geometry-inspired methodologies (Schug et al., 2005; Pan et al., 2012).

We have contributed to these efforts with a fast and parameter-free bioinformatics tool (MGFM) to detect marker genes from microarray data (El Amrani et al., 2015). Here, we investigate whether the algorithm behind this tool can be applied to datasets derived from RNA sequencing (RNA-seq) and its single cell versions (scRNA-seq), which are being extensively applied in biomedical research for the genome-wide evaluation of gene expression levels. One could expect that RNA-seq has several advantages over DNA microarrays for marker identification due to its higher sensitivity for general transcript detection (Zhao et al., 2014). The Marker Gene Finder in RNA-seq data (MGFR) calculates a score that indicates the specificity of each gene to each sample type (El Amrani et al., 2015). In addition, MGFR can map gene identifiers to gene symbols and Entrez Gene IDs, which is done with the R package biomaRt (Durinck et al., 2005).

We applied MGFR to six RNA-seq datasets that characterize the expression profiles of different cell and tissue types. Then, we evaluated whether the use of MGFR’s marker genes resulted in better clustering of the samples than using the complete set of genes, according to their reported cell or tissue labels. Next, we compared these results with those of other five marker detectors: the Specificity Measure (SPM) (Pan et al., 2012); the z-score (Kitsak et al., 2016); two classical DGEA approaches based on pairwise *t*- and Wilcoxon rank sum tests (Lun, McCarthy & Marioni, 2016); and a baseline detector that produces a random list of marker genes per sample type (see the Methods for details). In addition, we contrasted the markers identified by MGFR and the other methods with gold-standard marker genes available for three of the analyzed datasets. Finally, we carried out Gene Ontology (GO) (Ashburner et al., 2000) and REACTOME (Fabregat et al., 2016) enrichment analyses of MGFR’s candidate markers to assess their functional relationship with their corresponding cell or tissue type.

MGFR is available as a Bioconductor R package (https://doi.org/doi:10.18129/B9.bioc.MGFR), which facilitates the access to this method and its integration to more complex bioinformatics pipelines.

## Materials & Methods

### Data sources

We analyzed six different transcriptomics datasets, three measured with standard RNA-seq and three with scRNA-seq (see Table 1).

**Table 1:** Datasets analyzed in this work.

**Name**	**Type**	**Units**	**Reported labels**
Primordial	scRNA-seq	counts	Primordial germ cells and somatic cells at 4- and 10-week gestation	GSE63818
Blastocyst	scRNA-seq	counts	Epiblast, primitive endoderm and trophectoderm	GSE66507
Embryo	scRNA-seq	RPKM	Oocyte, zygote, 2-, 4- and 8-cell embryo, morulae and late blastocyst	GSE36552
Myogenesis	RNA-seq	RLE	Myoblast, myocyte and myotube	FANTOM5
Immune	RNA-seq	TMM	B, CD4+ T, CD8+ T and NK cells, neutrophils and monocytes	GSE60424
Tissues	RNA-seq	FPKM	Adrenal, brain, bone marrow, colon, endometrium, esophagus, heart, kidney, liver, lung, lymph node, prostate, salivary gland, spleen, testis and thyroid	E-MTAB-1733

The *Primordial* dataset corresponds to the transcriptome of human primordial germ cells from the migrating to the gonadal stage, as well as their neighboring somatic cells (Guo et al., 2015). We selected female samples at 10 and 4 weeks of gestation and the somatic samples for the same time points.

The *Blastocyst* dataset profiles the transcriptome of the different cell types comprising the human blastocyst (epiblast, trophectoderm and primitive endoderm) (Blakeley et al., 2015), whereas the *Embryo* data covers all the stages of human pre-implantation development (oocyte, zygote, 2-cell, 4-cell, 8-cell, morulae and blastocyst) (Yan et al., 2013).

*Myogenesis* is a time course RNA-seq assay covering the differentiation of human primary skeletal myoblasts into myotubes (Arner et al., 2015). We selected healthy samples only and labeled day 0 as myoblasts, day 4 as myocytes and day 12 as myotubes according to (Bentzinger, Wang & Rudnicki, 2012).

The *Immune* dataset compares the transcriptomes of 6 immune cell types (Linsley et al., 2014). We selected three samples from healthy subjects for each of the six immune cell types: neutrophils, monocytes, B cells, CD4+ T cells, CD8+ T cells, and natural killer cells (see Table S1).

*Tissues* is a subset of an RNA-seq assay aimed at classifying the tissue-specific expression of genes across a representative set of human tissues (Fagerberg et al., 2014). We selected 16 tissues from this dataset: adrenal, bone marrow, brain, colon, endometrium, esophagus, heart, kidney, liver, lung, lymph node, prostate, salivary gland, spleen, testis, and thyroid.

### Data preprocessing

The *Primordial* and *Blastocyst* count data were size factor normalized using DESeq2 (Love, Huber & Anders, 2014), followed by log transformations (log_2_(*X* + 1)). Only genes with expression values in at least two samples were considered. Drop-out events were imputed with DrImpute (Gong et al., 2018).

The normalized versions of the *Embryo* and *Myogenesis* datasets were downloaded from https://www.nature.com/articles/nsmb.2660#supplementary-information and http://fantom.gsc.riken.jp/5/sstar/Myoblast_to_myotube_(wt_and_DMD), respectively. Gene expression in the former was given by reads per kilobase per million mapped reads (RPKM) and in the latter by relative log expression (RLE). The *Immune* data were also already normalized and downloaded from https://www.ncbi.nlm.nih.gov/geo/query/acc.cgi?acc=GSE60424. The gene expression units were edgeR’s trimmed mean of M-values (TMM) (Robinson, McCarthy & Smyth, 2010). We log_2_-transformed the expression values of these three datasets after adding a pseudo-count of 1.

The raw reads from the *Tissues* study were mapped to the GRCh37 version of the human genome with Tophat v2.1.0 (Trapnell, Pachter & Salzberg, 2009). FPKM (fragments per kilobase of exon model per million mapped reads) values were calculated using cuffnorm v2.2.1 (Trapnell et al., 2010) and log_2_ transformed after adding a pseudo-count of 1. The used data were extracted after processing of all samples and averaging across technical replicates.

### Identification of marker genes

The algorithm underlying MGFR is described in the first section of the Results and in our previous study (El Amrani et al., 2015). SPM represents the expression of a gene in sample *i* with an *s*-dimensional vector (0, 0, …, *x*_*i*_, …, 0, 0) and calculates its dot product with the vector representing the gene’s expression profile (*x*_1_, *x*_2_, …, *x*_*i*_, …, *x*_*s*_). The *z*-score computes the significance of gene expression in sample *i* as *z*(*i*) = (*x*_*i*_ − *μ*(*x*))∕*σ*(*x*) with *μ*(*x*) and *σ*(*x*) the mean and standard deviation of the gene expression profile, respectively.

For the pairwise *t*- and Wilcoxon rank sum tests, we used functions findMarkers, pairwiseWilcox and combineMarkers from the R package scran v1.10.2 (Lun, McCarthy & Marioni, 2016) with parameters pval.type = “all” and direction = “up”. findMarkers runs function pairwiseTTests and passes the result to combineMarkers. The latter function consolidates the DGEA results from any pairwise comparison between sample types into a single list of up-regulated genes for each type. To do so, a test for the null hypothesis that a gene is not differentially expressed in all contrasts is performed and a *p*-value per gene, per sample type is computed. This approach points at genes that are uniquely differentially expressed in each sample type (candidate markers). For more details we refer the reader to http://bioconductor.org/packages/release/bioc/manuals/scran/man/scran.pdf.

We focused on genes with *p*-values ≤0.05. For the other marker detectors, we employed the specificity cutoffs suggested by their authors: *z*-scores ≥1 (Kitsak et al., 2016) and SPMs ≥0.4 (Pan et al., 2012).

### Gold-standard marker genes

We took the 45 reference markers for the *Blastocyst* dataset from a study of the three cell lineages of the human blastocyst by scRNA-Seq (Blakeley et al., 2015). For the *Immune* data, we collected a total of 71 known marker genes for the six examined cell types (see Table S2). Finally, for the *Tissues* dataset we considered the 2,500 markers reported in the TiGER database (Liu et al., 2008), which were available for ten human tissues only (bone marrow, brain, heart, kidney, liver, lung, lymph node, prostate, spleen, and testis). We provide these gold-standard lists of markers in File S1.

AUPRCs an AUROCs were computed with the R package precrec v0.9.1 (Saito & Rehmsmeier, 2017).

### Functional enrichment analyses

Functional enrichment analyses were carried out with the R package FunEnrich (https://github.com/galanisl/FunEnrich), using the complete set of genes in each expression matrix as background. The resulting *p*-values were corrected for multiple testing with the Benjamini–Hochberg method and the top five most significant GO and REACTOME terms are shown in Figs. S4–S6.

## Results

### Marker Gene Finder in RNA-seq data (MGFR)

MGFR expects a reference matrix *X* with the normalized expression values for *n* genes (rows) across *s* samples (columns), representing a total of *t* sample types (where *t* < *s*), and with at least two replicates for each type. Note that *X* has to be normalized such that between-sample comparisons are possible. Then, MGFR proceeds as follows:

**Figure 1:**
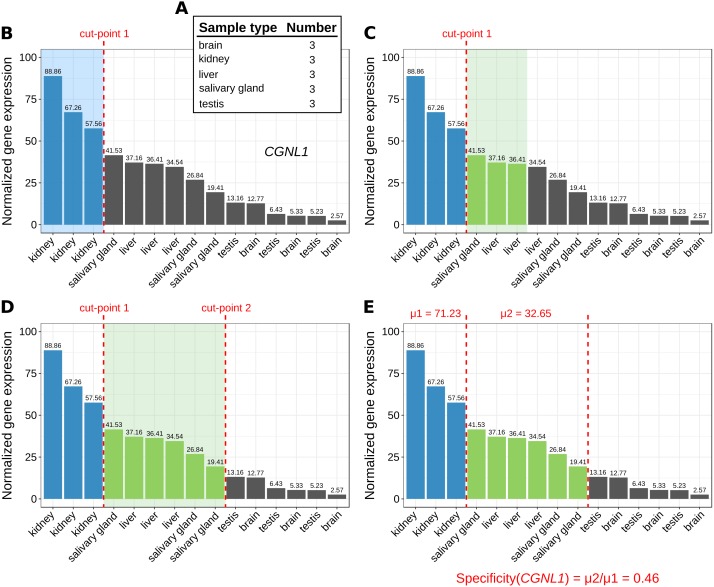
Marker identification with MGFR. An example showing the steps (A–E) that MGFR follows to identify marker genes. See text for details. The expression profile corresponds to gene *CGNL1* (cingulin like 1). For simplicity, only five tissues are shown.

1. The number of replicates per sample type are computed (Fig. 1A).
2. For each gene, its corresponding expression values across samples are sorted decreasingly (Fig. 1B).
3. Based on the sample type to which the highest gene expression value belongs, MGFR takes as many values from the expression profile as there are replicates for this type (see cut-point 1 in Fig. 1B). If all the values before the cut-point come from the same sample type and are ≥1, the gene is a potential marker and MGFR proceeds to step 4. Otherwise, the gene is discarded and the process restarts from step 2.
4. MGFR seeks to define a second cut-point to assign a specificity score to the candidate marker. For this, it checks the sample type of the expression value that is right next to cut-point 1 and takes as many values from the expression profile as there are replicates for this type (Fig. 1C). If all the considered values belong to the same sample type, cut-point 2 is defined. Otherwise, MGFR keeps taking values until all replicates of the types being considered are covered (Fig. 1D).
5. Finally, the specificity score is calculated as the ratio of the average expression of samples between the first and second cut-point divided by that of samples preceding the first cut-point. This score has a value between 0 and 1. A score near 0 would indicate high specificity and a large score, closer to 1, would indicate low specificity (see Fig. 1E).

The cutoff value of 1 (used in step 3) is justified by previous studies on appropriate thresholds for gene expression calling (Hebenstreit et al., 2011; Wagner, Kin & Lynch, 2013). In addition to this cutoff, the main change in MGFR compared to MGFM is the mapping of gene identifiers to gene symbols and Entrez Gene IDs, which is done using the R package biomaRt (Durinck et al., 2005). MGFR supports Ensembl, RefSeq, and UCSC identifiers. More details about the method are also given in the vignette of the R package.

### MGFR’s markers accurately characterize the functional identity of different cell and tissue types

Given the expression matrix *X*, MGFR produces a list with *t* elements, each one containing the set of markers that better differentiates between the *t* sample types contained in *X*. If we use the union of these sets of markers as features for the *s* samples in *X*, the application of a clustering algorithm should result in a better grouping of the samples into *t* clusters than using the full set of *n* genes. Under this rationale, we applied MGFR to six different RNA-seq datasets, three single cell and three standard assays (see the Methods and Table 1). Then, we used Principal Component Analysis (PCA) to cluster the *s* samples in each reduced matrix *X*_*MGFR*_, which only contains expression values for the *n*_*MGFR*_ marker genes, where *n*_*MGFR*_ <  < *n*. Finally, to determine cluster memberships for each sample, we input the obtained first two principal components to the k-means algorithm, using *k* equal to the reported number of cell or tissue types in each dataset (see Table 1). We quantified clustering quality with three different metrics (Normalized Mutual Information or NMI, Purity and Adjusted Rand index (Manning, Raghavan & Schütze, 2008)). Since k-means always starts with *k* random centroids, we averaged the quality metrics of 100 clustering results. The same procedure was applied to the other five marker detectors mentioned in the Introduction (see the Methods for details). For the random detector, we repeated the experiment 10 times.

**Figure 2:**
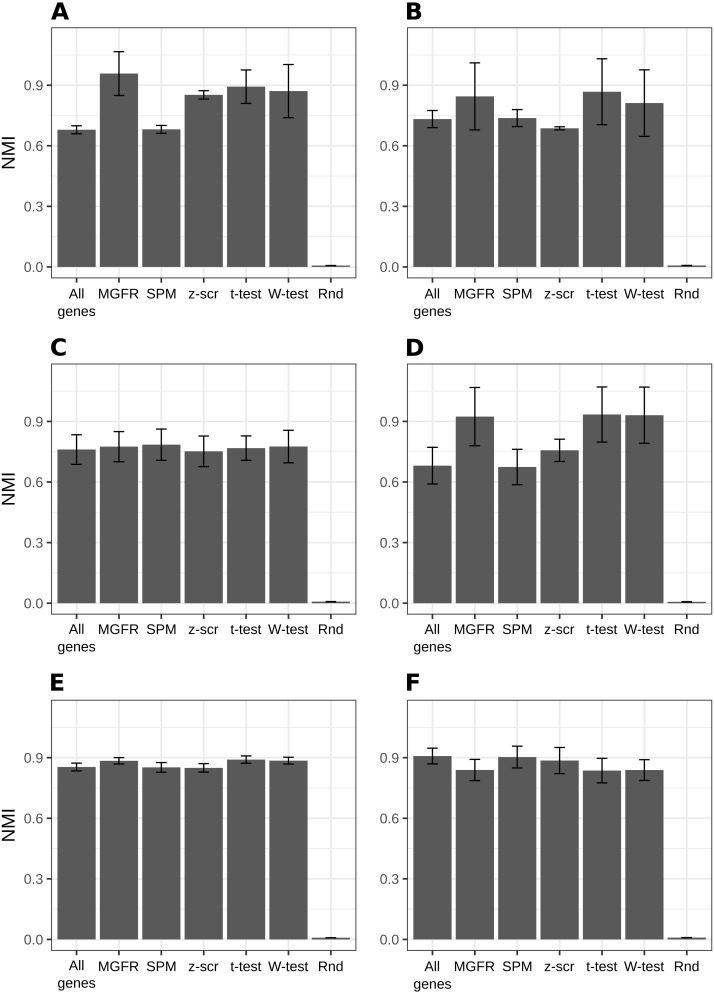
Benchmarking MGFR against other marker detectors. The biomarkers identified by MGFR lead to clustering results that are as good as or better than those achieved by other methods according to the NMI metric (SPM: Specificity Measure, *z*-scr: *z*-score, *t*-test: pairwise *t*-tests; Wilcoxon-test: pairwise Wilcoxon rank sum tests, Rnd: Random). Error bars correspond to standard deviations. (A) *Primordial*, (B) *Blastocyst*, (C) *Embryo*, (D) *Myogenesis*, (E) *Tissues* and (F) *Immune* datasets.

**Figure 3:**
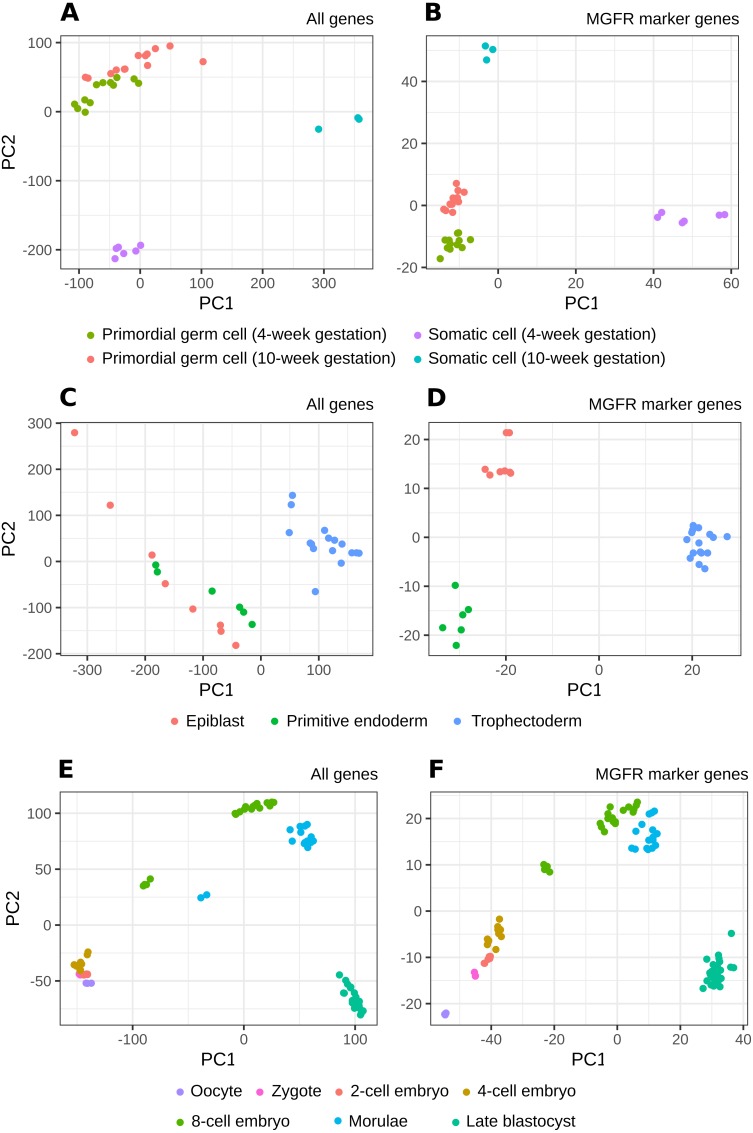
PCA results of scRNA-seq datasets. The clusters resulting from applying PCA to the complete expression matrix *X* (A, C, E) or to the reduced one *X*_*MGFR*_ (B, D, F) from the three scRNA-seq datasets: (A, B) *Primordial*, (C, D) *Blastocyst* and (E, F) *Embryo*.

**Figure 4:**
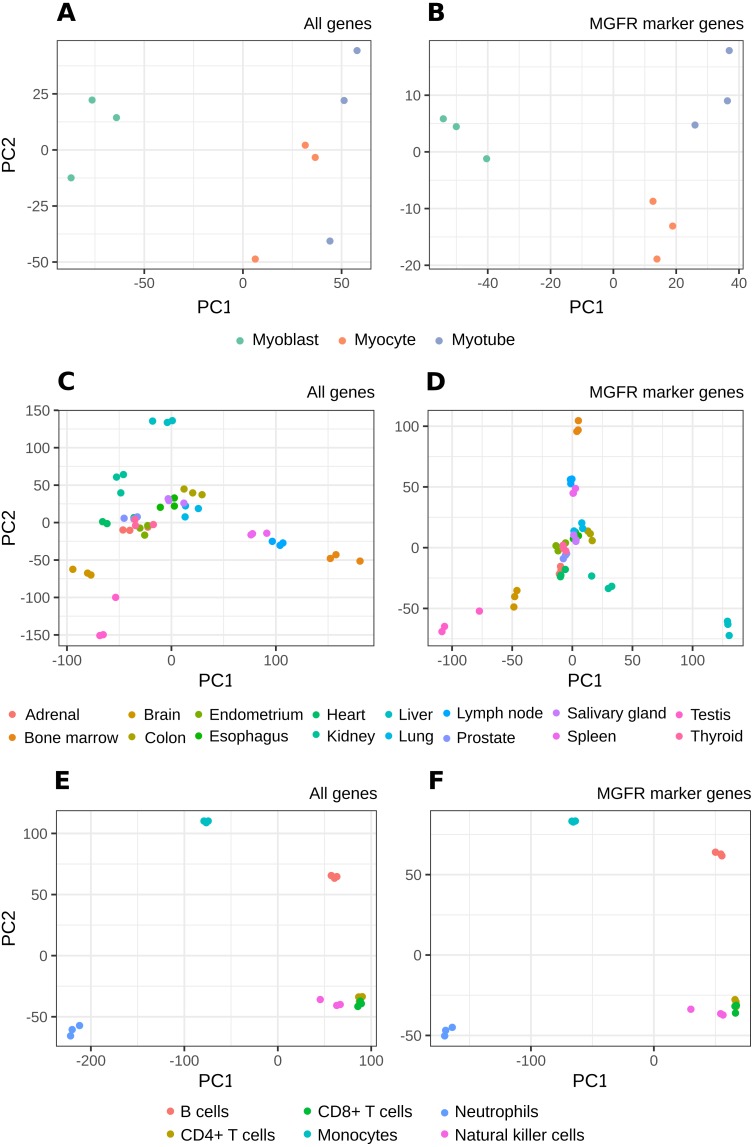
PCA results of RNA-seq datasets. The clusters resulting from applying PCA to the complete expression matrix *X* (A, C, E) or to the reduced one *X*_*MGFR*_ (B, D, F) from the three RNA-seq datasets: (A, B) *Myogenesis*, (C, D) *Tissues* and (E, F) *Immune*.

Figure 2 and Figs. S1–S2 show that MGFR performs as good as or better than the other five approaches in spite of its simplicity. This suggests that our proposed method is able to detect the set of markers that more strongly emphasizes the differences between sample types. This effect is more clearly illustrated in Figs. 3 and 4, which highlight a better separation between cell and tissue types in PCA space if the reduced expression matrix *X*_*MGFR*_ is used instead of the original one containing all genes. It is important to note that Figs. 3 and 4 illustrate the effect of using the markers detected by MGFR in the clustering of the data, but markers selected by other methods would also result in good separation in the PCA. The next section evaluates the selection of markers obtained by MGFR and by other methods in further detail.

### MGFR identifies known cell and tissue markers

To further evaluate the performance of the tools benchmarked in Fig. 2, we compared their predicted markers with gold-standard lists available for three of the considered datasets: *Blastocyst*, *Immune* and *Tissues* (see Table 1, File S1 and the Methods for details).

In general, MGFR identified 6 of the 45 *Blastocyst* gold-standard markers, 58 of the 71 *Immune* ones and 1,343 of the 2,500 *Tissues* genes. We checked whether these overlaps were significantly larger than expected by chance using a list of 1,000 randomly generated marker sets of the same size as the actual ones and measuring the size of the intersection with the gold-standard marker sets. The random distribution of overlaps was compared with the actual values via a z-test, which resulted in a *p*-value of 6.84 × 10^−38^ for *Blastocyst*, 1.76 × 10^−12^ for *Immune* and 4.68 × 10^−261^ for *Tissues*. We provide MGFR’s predicted markers in File S2.

**Figure 5:**
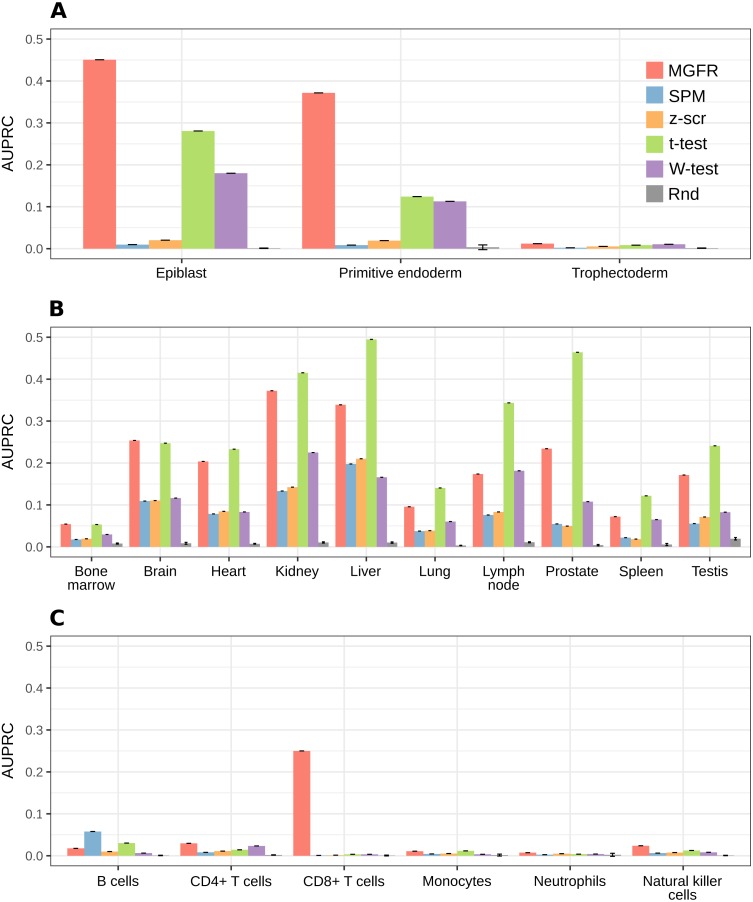
MGFR assigns better specificity scores to known markers. MGFR’s specificity score allows for a more precise discrimination between good and poor candidate markers, without sacrificing as much sensitivity (recall) as with the other marker detectors. (A) *Blastocyst*, (B) *Tissues* and (C) *Immune* datasets.

For a more systematic comparison between marker detectors, the per-sample-type list of markers identified by each method was sorted from more to less specific, based on the marker specificity scores. Then, the list was scanned from top to bottom using each score as a cutoff to determine which genes were considered markers (above the cutoff) and which were not (below the cutoff). We compared the candidate markers at each cutoff against the gold-standard list for the sample type and computed precision (fraction of candidate markers that are actually markers) and recall (fraction of all actual markers that has been considered so far) to construct a precision–recall curve and finally use the area under it (AUPRC) as a means to benchmark the detectors. Figure 5 highlights that MGFR outperforms all the other methods in the *Blastocyst* and *Immune* datasets and performs comparably to the t-tests in the *Tissues* dataset. The only exception is the B cells in the *Immune* data, where SPM is able to assign better scores to the markers of this cell type. Whenever possible, we also constructed Receiver Operating Characteristic (ROC) curves and computed the area under them (AUROC) to complement the AUPRC results (see Fig. S3).

### MGFR’s novel tissue and cell markers are biologically meaningful

To assess whether MGFR’s markers that are not reported in our gold-standard lists are functionally related to their corresponding cell or tissue type, we performed functional enrichment analyses (see ‘Methods’ for details).

In Figs. S4–S6, we can see that the most enriched GO terms and REACTOME pathways demonstrate that many of the predicted marker genes for the examined cell types and tissues have functions consistent with these sample types. For example, the predicted markers for the Trophectoderm are enriched in *transmembrane transport* processes that are typical of placental cells (see Fig. S4), candidate markers of B cells are enriched in the *positive regulation of B cell activation* and *antigen binding* (see Fig. S5), markers of Neutrophils are enriched in *neutrophil degranulation* and the *innate immune system* (see Fig. S5), those for the brain are enriched in *chemical synaptic transmission* and the *neuronal system* (see Fig. S6) and markers of the testis are enriched in *spermatogenesis* (see Fig. S6).

Interestingly, some of the markers that MGFR predicted for the *Tissues* dataset (see File S2) have been recently proposed as novel marker genes, such as *TMEM72* in kidney, *RTP3* (also known as *TMEM7*), *SRHC*, *TTC36* (also known as *HBP21*), *TNFAIP8L1*, and *ETNPPL* in liver, and *RTKN2* in lung. Wrzesiński et al. (2015) reported a downregulation of *TMEM72* in clear cell renal cell carcinoma. In recent studies, *RTP3* (Zhou et al., 2007), *SRHC* (Zheng et al., 2015), *TTC36* (Jiang et al., 2015), *TNFAIP8L1* (Zhang et al., 2015), and *ETNPPL* (Ding et al., 2016) were reported to be downregulated in hepatocellular carcinoma. Also, *RTKN2* was reported as novel candidate marker gene for idiopathic interstitial pneumonias (Steele et al., 2015). The expression of these genes was found to be downregulated in diseased tissues as compared to normal tissues. Hence, we hypothesize that these disease-implicated genes in tissue-specific diseases may play important roles in the function of normal tissues.

## Discussion

We benchmarked MGFR against existing marker detectors and showed that it is able to detect the genes that most clearly discriminate between sample types. Also, these candidate markers significantly overlap with gold-standard marker lists. Furthermore, MGFR’s specificity score outperformed the scores produced by other methods in AUPRC benchmarks, which means that the genes with the top MGFR scores are in agreement with known tissue and cell markers. The functional enrichment analysis of candidate markers that are not reported in the literature showed that they are part of processes and pathways consistent with their associated tissue or cell type.

Importantly, the gold-standard lists that we used may not be complete or contain false positives. For example, the gold-standard marker genes for the *Tissues* dataset were extracted from TiGER (see ‘Methods’) and calculated based on expressed sequence tag counts (EST). The use of ESTs to quantify gene expression levels is less sensitive than RNA-seq technology. Therefore, the lists from TiGER are not comprehensive, and marker genes predicted with MGFR may not be contained in the lists from TiGER. Also, in the case of the *Immune* dataset, the surface markers that we considered are valid at the protein level but the transcripts from the corresponding genes may not be present in the different immune cell types.

It is worth noting that the list of predicted marker genes for a sample type depends on the number of samples included in the reference dataset, and this may differ when adding a new sample type to the expression matrix. Note also that the number of markers obtained for a dataset will depend on the differences in expression. That is, for sets with categories with very similar expression one would expect to obtain fewer markers. Thus, our method identifies markers in the context of the samples provided by the researcher. These markers are not supposed to be universal in the sense of being specific of the cell or tissue versus all other cell and tissue types in the organism, but reflect the distribution of samples of interest. This can be an advantage when the researcher is trying to find differences in a local environment, like, for example, a collection of cell types from an organ, and our benchmarks suggested that MGFR’s markers allow for a better separation of the data by sample type (see Figs. 3 and 4).

One limitation of MGFR is that when the number of replicates per sample type is very large, the probability that they group together in step 2 of the algorithm is low (see Fig. 1). This means, however, that MGFR is particularly useful when there are only a few replicates per sample type and DGEA lacks the statistical power to detect meaningful markers (Yu, Fernandez & Brock, 2017). We also note that the analyzed datasets were normalized with different approaches, which highlights that our algorithm should work with any RNA-seq quantification method, provided the data is properly processed and normalized such that expression values can be compared between samples. One possible extension of MGFR would be to sort genes based on non-parametric ranks rather than gene expression (see Fig. 1B). This would eliminate the need for between-sample normalization before MGFR is applied to a gene expression matrix.

## Conclusions

We have previously developed a bioinformatics tool (MGFM) for marker gene detection from microarray data. In this work, we present an adaptation that enables the detection of marker genes from RNA-seq data. In contrast to very comprehensive but static databases of tissue-specific genes such as Tissue-specific Gene Expression and Regulation (TiGER) (Liu et al., 2008), our tool enables users to easily modify and adapt the reference set of genes to their set of interest. Furthermore, MGFR might be applied to identify novel candidate marker genes.

The tool is provided as a Bioconductor package called MGFR (https://doi.org/doi:10.18129/B9.bioc.MGFR) and will be integrated into the CellFinder platform (http://cellfinder.org) and connected to its molecular database, which will serve as a data source. CellFinder (Stachelscheid et al., 2014) is a comprehensive online resource for diverse data, characterizing mammalian cells in different tissues and development stages. It is built from carefully selected datasets stemming from other curated databases and the biomedical literature.

##  Supplemental Information

10.7717/peerj.6970/supp-1 Supplemental Information 1 Supplementary document with Figures S1-S6 and Tables S1 and S2

10.7717/peerj.6970/supp-2 File S1 Gold-standard lists of markers for three of the considered datasets: Blastocyst, Immune and Tissues

10.7717/peerj.6970/supp-3 File S2 Markers predicted by MGFR for the Blastocyst, Immune and Tissues datasets

## Additional Information and Declarations

### Data Availability

The following information was supplied regarding data availability: The Bioconductor package is available at: 10.18129/B9.bioc.MGFR.
