# Supplementary material for: Detection of condition-specific marker genes from RNA-seq data with MGFR
Source: PeerJ. 2019 May 27;7:e6970. doi: 10.7717/peerj.6970 (PMC6542349; doi:10.7717/peerj.6970)
Supplement: Supplemental Information 1 [file peerj-07-6970-s001.pdf]

SUPPLEMENTARY INFORMATION

Detection of condition-specific marker genes from RNA-seq data with MGFR

Khadija El Amrani[1], Gregorio Alanis-Lobato[2], Nancy Mah[1], Andreas Kurtz[1], and Miguel A. Andrade-Navarro[*,2]

[1] *Charité - Universitätsmedizin Berlin, Berlin Brandenburg Center for Regenerative Therapies (BCRT), 13353 Berlin, Germany*
[2] *Faculty of Biology, Johannes Gutenberg Universität, Biozentrum I, Hans-Dieter-Hüsch-Weg 15, 55128 Mainz, Germany*

[*] Correspondence should be addressed to andrade@uni-mainz.de

Supplementary Figures

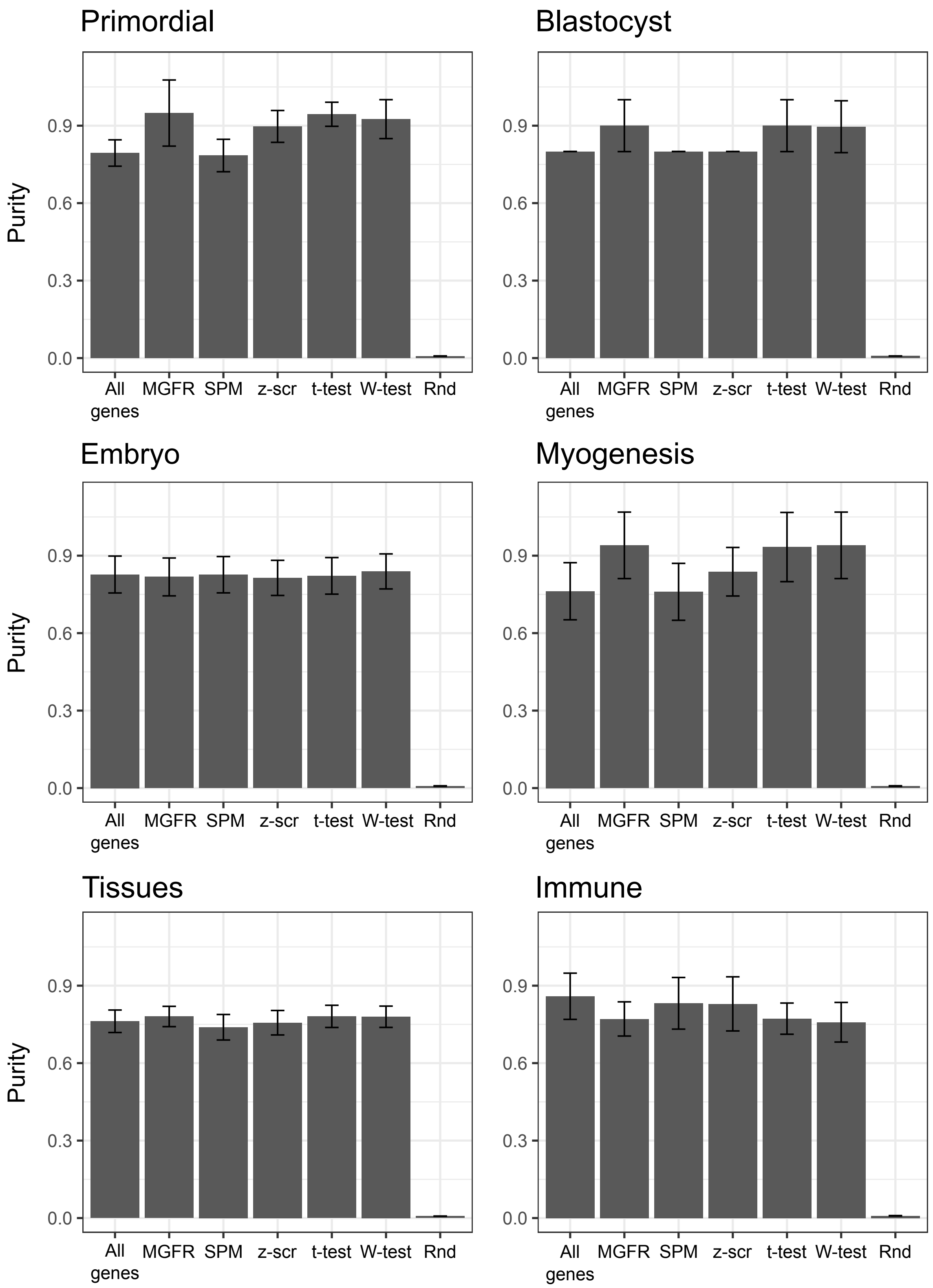

Figure S1: Benchmarking MGFR against other marker detectors (Purity). The biomarkers identified by MGFR lead to clustering results that are as good or better than those achieved by other methods (SPM: Specificity Measure, z-scr: z-score, t-test: Combination of pairwise t-tests, W-test: Combination of pairwise Wilcoxon rank sum tests, Rnd: Random). Error bars correspond to standard deviations.

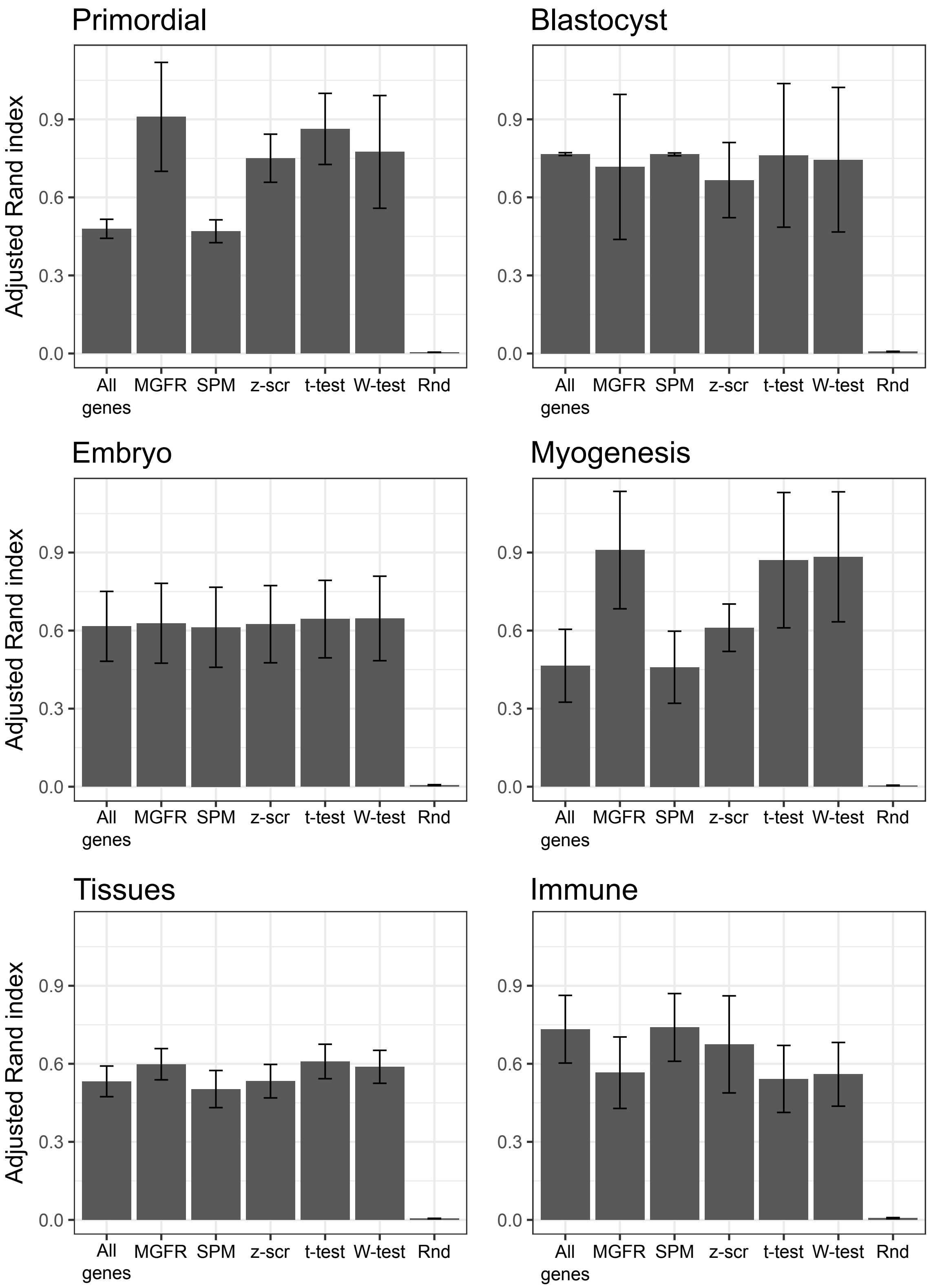

Figure S2: Benchmarking MGFR against other marker detectors (Adjusted Rand index). The biomarkers identified by MGFR lead to clustering results that are as good or better than those achieved by other methods (SPM: Specificity Measure, z-scr: z-score, t-test: Combination of pairwise t-tests, W-test: Combination of pairwise Wilcoxon rank sum tests, Rnd: Random). Error bars correspond to standard deviations.

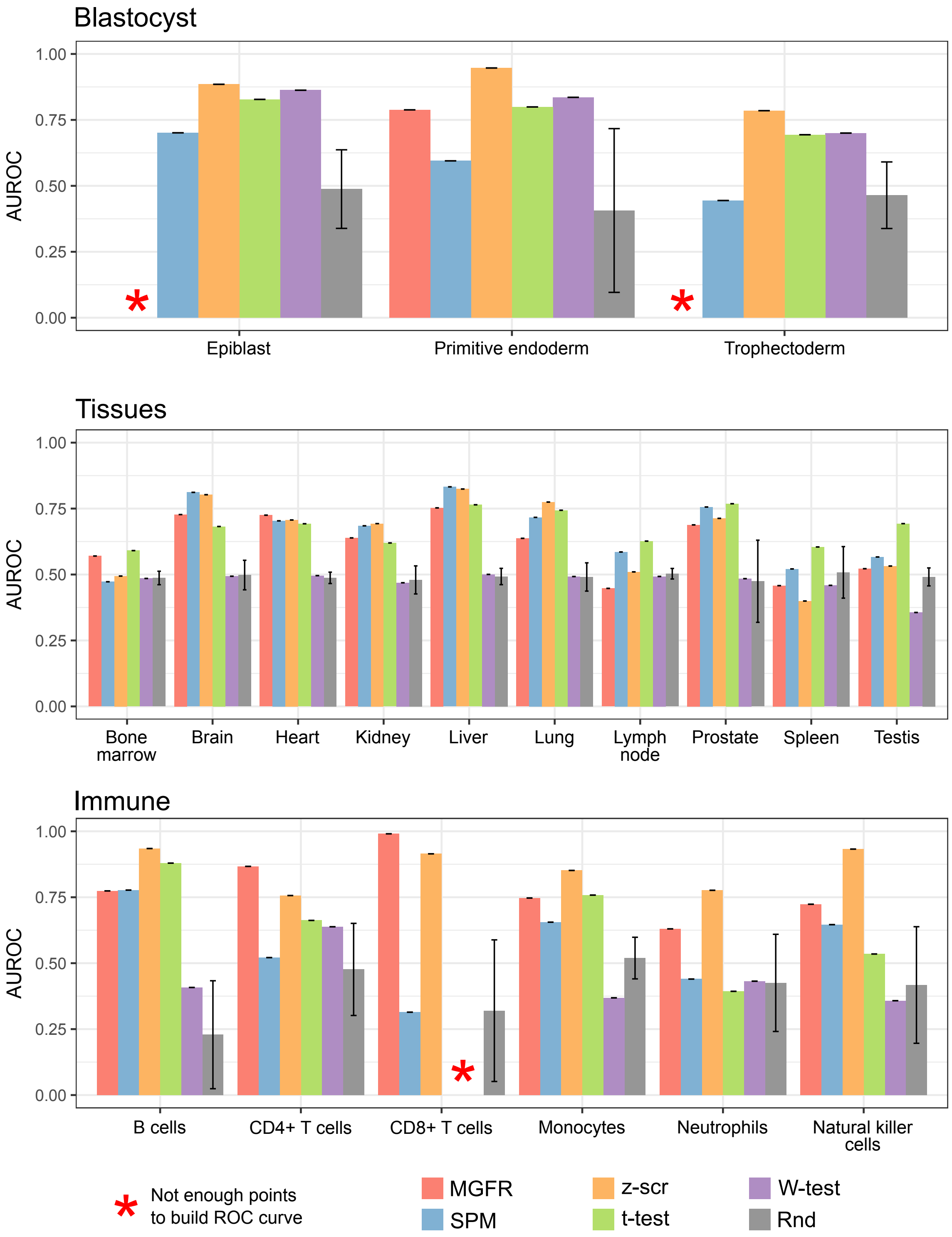

Figure S3: Performance evaluation based on identification of known markers. MGFR is as good or better than other methods at identifying known biomarkers when the Area Under the ROC curve (AUROC) metric is employed. Cases in which marker detectors did not produced enough candidates to build a ROC curve are marked with a red star.

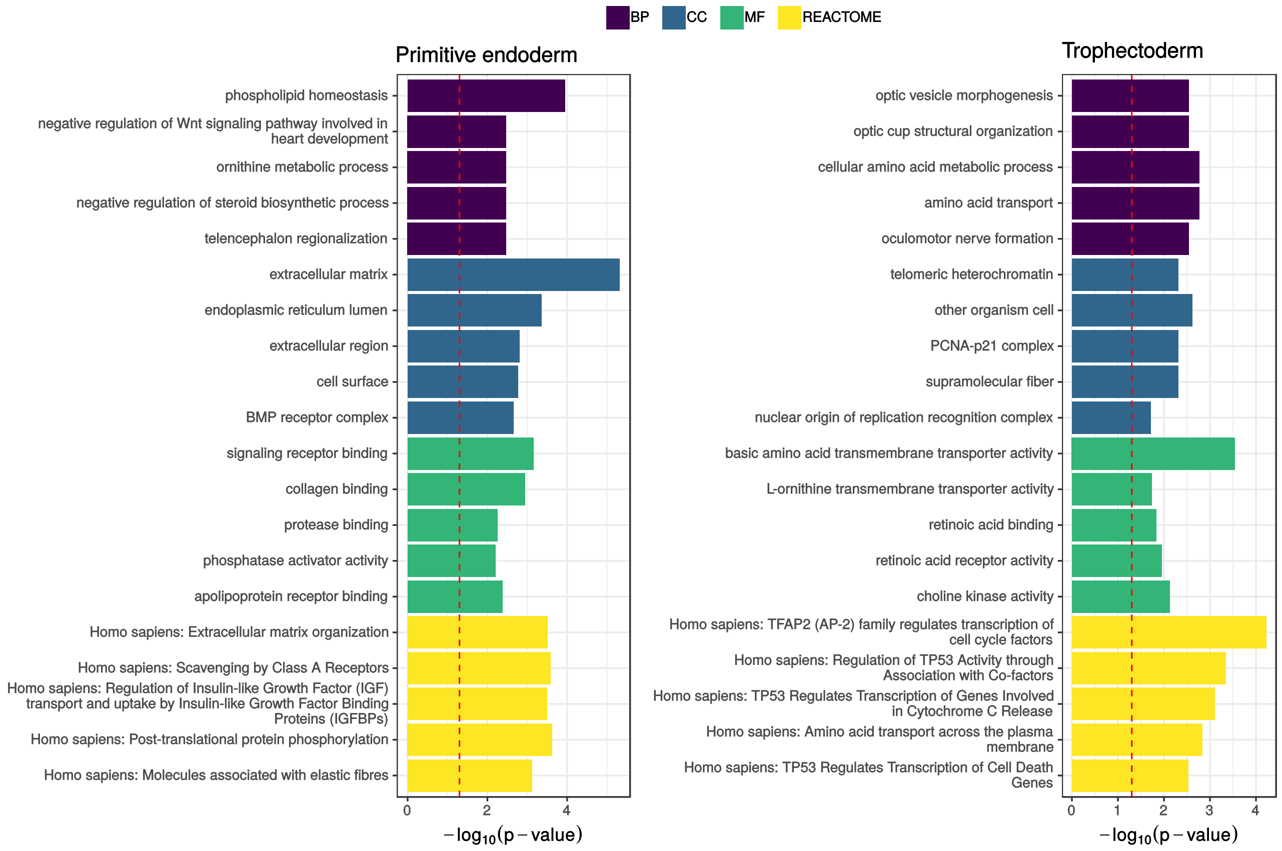

Figure S4: Functional enrichment analysis of MGFR's novel markers (Blastocyst). Functional enrichment analysis of MGFR's candidate markers that are not reported in the literature. We considered the three aspects of the Gene Ontology (Biological Process or BP, Cellular Compartment or CC and Molecular Function or MF) and REACTOME pathways. The Epiblast was not considered because the number of markers predicted by MGFR was too small for this kind of analysis.

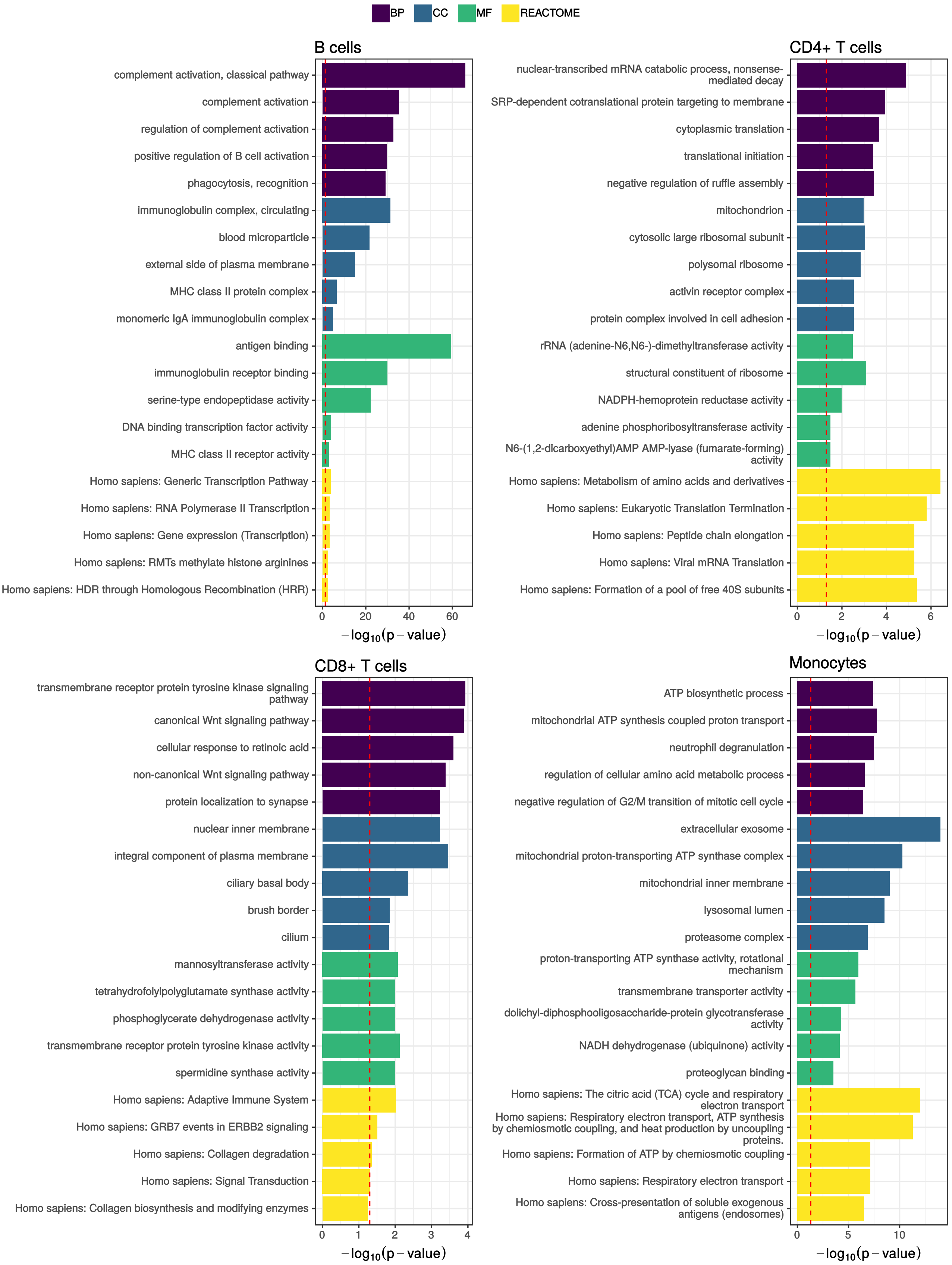

Figure S5: Functional enrichment analysis of MGFR's novel markers (Immune). Functional enrichment analysis of MGFR's candidate markers that are not reported in the literature. We considered the three aspects of the Gene Ontology (Biological Process or BP, Cellular Compartment or CC and Molecular Function or MF) and REACTOME pathways.

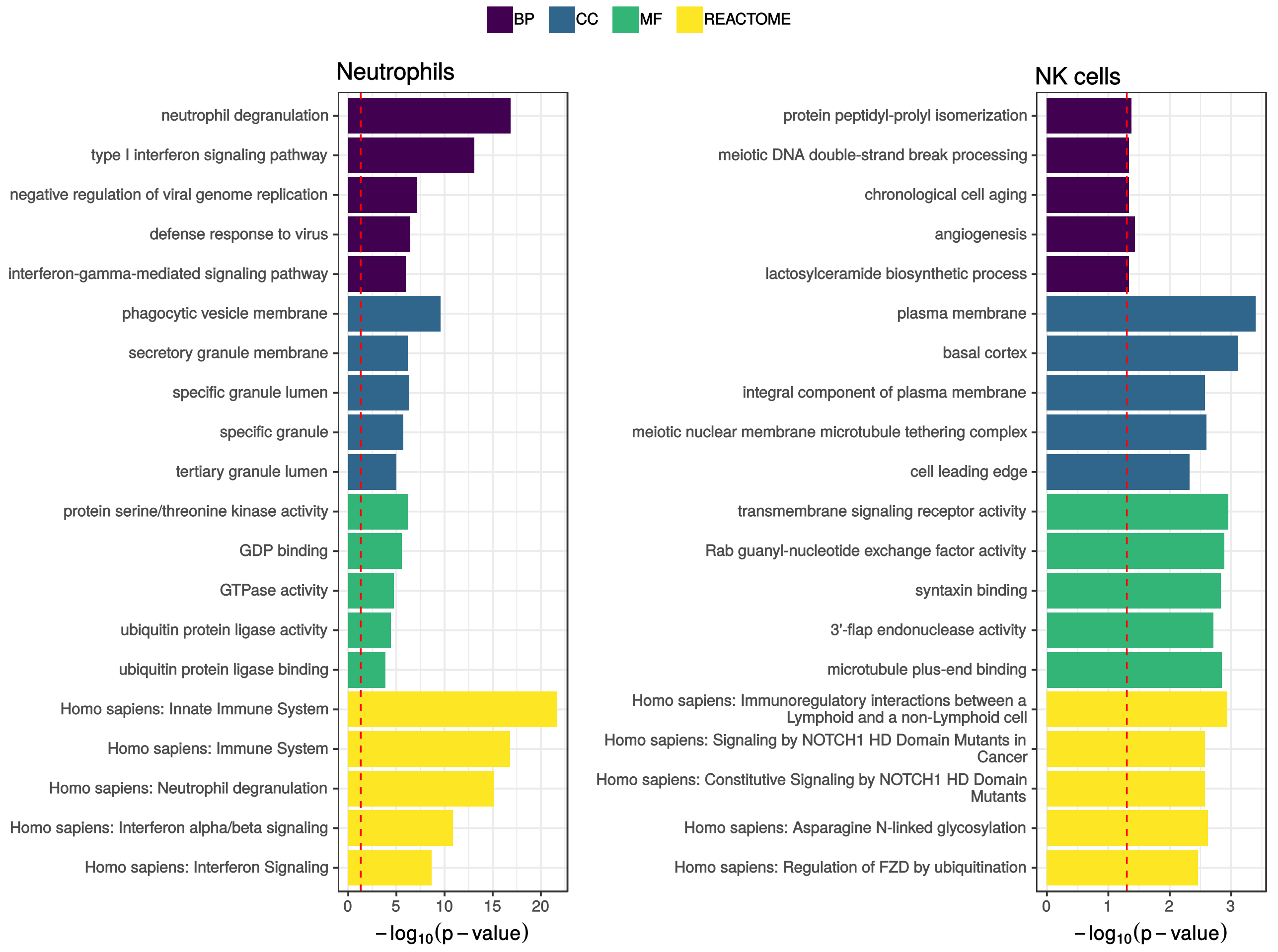

Figure S5 (Cont.): Functional enrichment analysis of MGFR's novel markers (Immune). Functional enrichment analysis of MGFR's candidate markers that are not reported in the literature. We considered the three aspects of the Gene Ontology (Biological Process or BP, Cellular Compartment or CC and Molecular Function or MF) and REACTOME pathways.

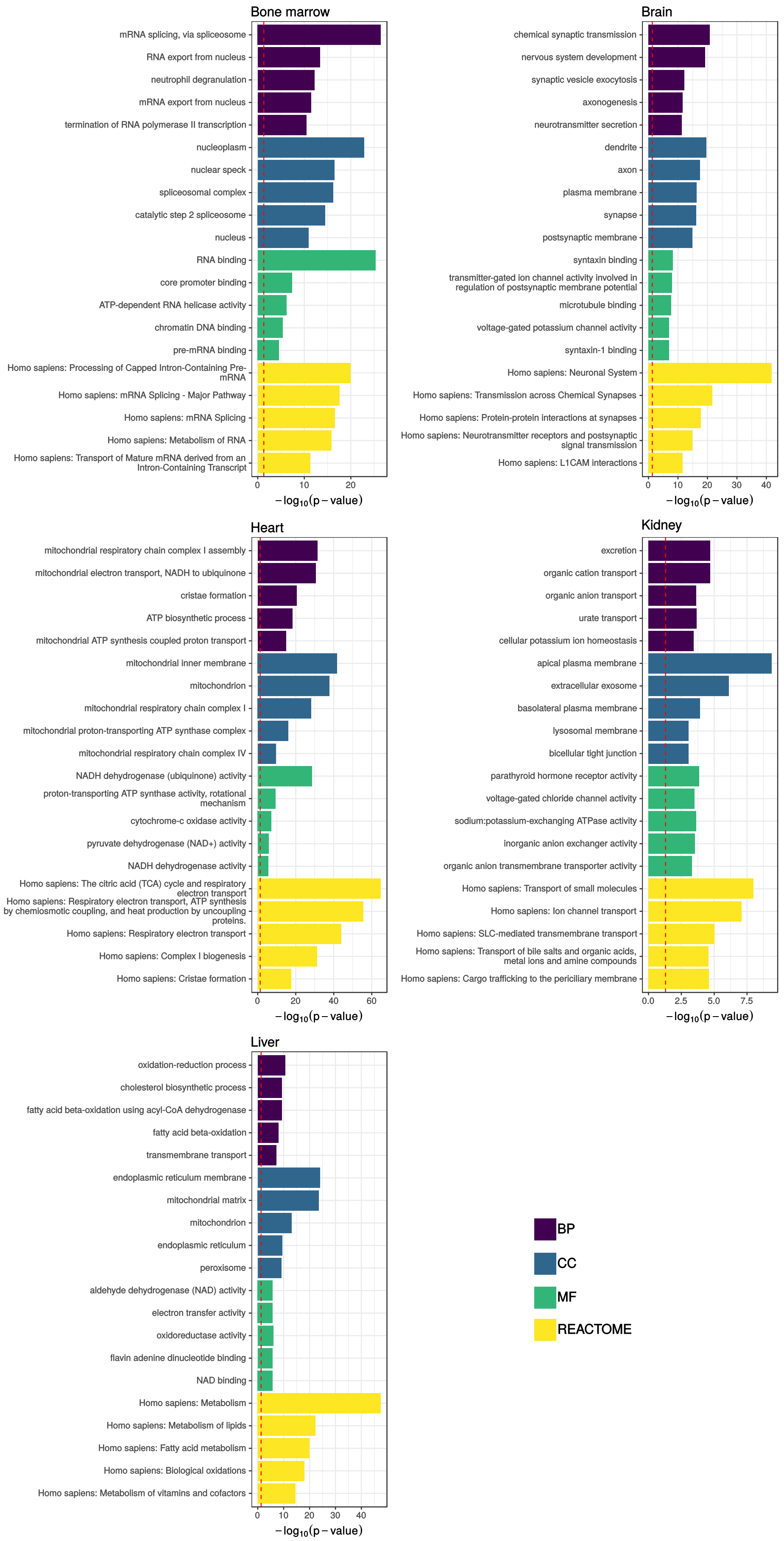

Figure S6: Functional enrichment analysis of MGFR's novel markers (Tissues). Functional enrichment analysis of MGFR's candidate markers that are not reported in the literature. We considered the three aspects of the Gene Ontology (Biological Process or BP, Cellular Compartment or CC and Molecular Function or MF) and REACTOME pathways.

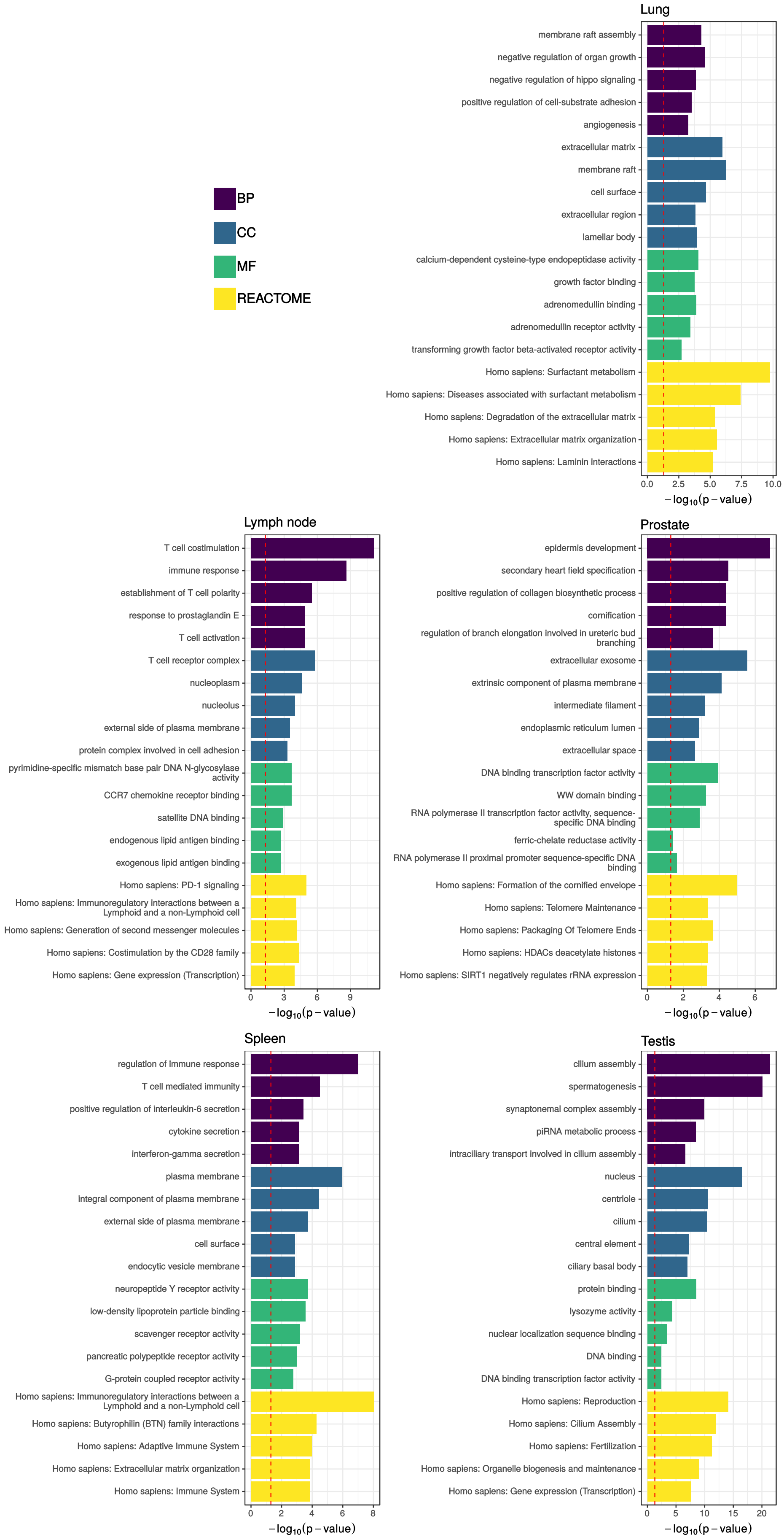

Figure S6 (Contd.): Functional enrichment analysis of MGFR's novel markers (Tissues). Functional enrichment analysis of MGFR's candidate markers that are not reported in the literature. We considered the three aspects of the Gene Ontology (Biological Process or BP, Cellular Compartment or CC and Molecular Function or MF) and REACTOME pathways.

Supplementary Tables

Cell type	Sample IDs
Neutrophils	lib288, lib295, lib226
CD4+ Cells	lib291, lib298, lib312
CD8+ Cells	lib230, lib313, lib299
NK Cells	lib231, lib293, lib300
B Cells	lib290, lib297, lib311
Monocytes	lib289, lib310, lib296

Table S1: The corresponding IDs for the samples used in the immune cell dataset

Cell type	Marker genes	Reference	Mean spec. score*
Neutrophils	***MME***, ***ITGAX***, ***ANPEP***, ***FCGR3A***, ***FCGR2A***, ***SELL***, ***FCGR1A***, ***CEACAM8***, ***C5AR1***, ***CSF3R***, ***CXCR1***, ***CXCR2***, ***JAML***, ***TLR2***, *ITGAM*	[1]	0.15
CD4+ Cells	***CCR4***, ***CCR10***, ***CD40LG***, ***ICOS***, *CD4*, *IFNGR1*, *CXCR3*, *CCR5*, *IL18R1*, *LTBR*, *HAVCR2*, *CCR3*, *CCR8*, *PTGDR2*, *HAVCR1*, *KLRB1*, *CCR6*, *CD84*, *IL6R*, *SLAMF1*, *CXCR5*, *TNFSF4*, *PDCD1*	[1]	0.2
CD8+ Cells	***CD8***, *PTPRC*, *CCR7*, *CD28*	[2]	0.003
NK Cells	***KLRD1***, ***KIR2DL1***, ***KIR3DL1***, ***NCAM1***, ***CD244***, ***GZMB***, ***GNLY***, ***PRF1***, *KLRK1*	[3]	0.15
B Cells	***MS4A1***, ***CD79B***, ***CD79A***, ***CD22***, ***CD40***, ***CR2***, ***FCER2***, ***IGHM***, ***PAX5***, ***TNFRSF13B***	[3]	0.02
Monocytes	***CD14***, ***CD33***, ***FUT4***, ***LRP1***, ***CSF1R***, ***PVR***, ***CD163***, ***MSR1***, *ADAM10*, *CD93*	[4]	0.13

Table S2: The known marker genes used as gold-standard for the 6 immune cell types. Genes shown in bold were correctly identified by our tool MGFR. * The mean specificity score calculated by our tool for the correctly identified marker genes.

References

[1] Cell markers. https://www.biolegend.com/cell_markers. Accessed: 2018-04-18.

[2] T cell markers. http://www.abcam.com/primary-antibodies/effector-t-cell-markers. Accessed: 2018-04-17.

[3] Bradlee D. Nelms, Levi Waldron, Luis A. Barrera, Andrew W. Weflen, Jeremy A. Goettel, Guoji Guo, Robert K. Montgomery, Marian R. Neutra, David T. Breault, Scott B. Snapper, Stuart H. Orkin, Martha L. Bulyk, Curtis Huttenhower, and Wayne I. Lencer. CellMapper: rapid and accurate inference of gene expression in difficult-to-isolate cell types. *Genome Biology*, 17(1):201, dec 2016.

[4] Cd marker handbook. https://www.bdbiosciences.com/documents/cd_marker_handbook.pdf. Accessed: 2018-04-19.
